# Dr. George W. Lewis

**Published:** 1897-10

**Authors:** 


Dr. George W. Lewis, one of the oldest and best known homeopathic physicians in Buffalo, died August 30, 1897, aged 70 years. Dr. Lewis was a brother of Judge Loran L. Lewis, of this city, and he is survived by two sons, Dr. Geo. W. Lewis, Jr., and Edward E. Lewis. Dr. Lewis had been a prominent practitioner of medicine in Buffalo for many years, was a member of the several homeopathic medical societies—county, state and national—and was attending physician at the Buffalo Homeopathic Hospital.

